# A human programmed death-ligand 1-expressing mouse tumor model for evaluating the therapeutic efficacy of anti-human PD-L1 antibodies

**DOI:** 10.1038/srep42687

**Published:** 2017-02-16

**Authors:** Anfei Huang, Di Peng, Huanhuan Guo, Yinyin Ben, Xiangyang Zuo, Fei Wu, Xiaoli Yang, Fei Teng, Zhen Li, Xueming Qian, F. Xiao-Feng Qin

**Affiliations:** 1 Center of Systems Medicine, Institute of Basic Medical Sciences, Chinese Academy of Medical Sciences & Peking Union Medical College, Beijing, 200005, China; 2 Suzhou Institute of Systems Medicine, Suzhou, 215123, China; 3 Mabspace Biosciences (Suzhou) Co., Ltd, Suzhou, 215123, China; 4 Institutes of Biology and Medical Sciences, Soochow University, Suzhou, 215123, China

## Abstract

Huge efforts have been devoted to develop therapeutic monoclonal antibodies targeting human Programmed death-ligand 1 (hPD-L1) for treating various types of human cancers. However, thus far there is no suitable animal model for evaluating the anti-tumor efficacy of such antibodies against hPD-L1. Here we report the generation of a robust and effective system utilizing hPD-L1-expressing mouse tumor cells to study the therapeutic activity and mode of action of anti-human PD-L1 in mice. The model has been validated by using a clinically proven hPD-L1 blocking antibody. The anti-hPD-L1 antibody treatment resulted in potent dose-dependent rejection of the human PD-L1-expressing tumors in mice. Consistent with what have observed in autochthonous mouse tumor models and cancer patients, the hPD-L1 tumor bearing mice treated by anti-hPD-L1 antibody showed rapid activation, proliferation and reinvigoration of the cytolytic effector function of CD8^+^T cells inside tumor tissues. Moreover, anti-hPD-L1 treatment also led to profound inhibition of Treg expansion and shifting of myeloid cell profiles, showing bona fide induction of multilateral anti-tumor responses by anti-hPD-L1 blockade. Thus, this hPD-L1 mouse model system would facilitate the pre-clinical investigation of therapeutic efficacy and immune modulatory function of various forms of anti-hPD-L1 antibodies.

Recently monoclonal antibodies targeting immune checkpoint molecules have achieved unprecedented success in clinic for the treatment of a broad range of the most prevalent human cancers^1^,^2^,^3^,^4^. In particular, antibodies blocking the programmed death −1 (PD-1) /programmed death ligand-1 (PD-L1) pathway^1^,^3^,^4^,^5^ have demonstrated long-term durable and even complete clinical responses for a significant fraction of patients with a wide variety of advanced and highly refractory cancers^1^,^2^,^3^,^5^. Thus, there are vast medical needs for the development of highly effective and cost-saving therapeutic antibodies against PD-1 and PD-L1^1^,^3^,^5^.

PD-L1 was originally identified and cloned as a B7 family of co-stimulatory/co-inhibitory molecule, called B7-H1^6^, and subsequently determined to function primarily as a ligand for PD-1^7^. Survey of large panels of human and mouse tumor samples has revealed that PD-L1is highly expressed on tumor cells as well as host immune and stromal cells in the tumor microenvironment^1^,^4^,^6^. Interestingly, PD-L1 expression can be induced by many different cytokines, most prominently, by interferon gamma (IFN-g). As high PD-L1 expression in tumor tissues is often associated with the presence of infiltrating T cells (TILs) and IFN-g signature genes, it has been suggested that IFN-g produced by TILs is responsible for the induction of PD-L1 expression in the tumor microenvironment, which might be a mechanism of adaptive resistance exploited by tumor cells. In addition to immune-mediated induction, the loss of oncogenic phosphatase and tensin homolog (PTEN) and aberrant expression of epidermal growth factor receptor (EGFR) and nucleophosmin (NPM) /anaplastic lymphoma kinase (ALK) fusion protein has been reported to cause elevated PD-L1 expression in various tumors^4^. Furthermore, our own studies have recently shown that repression of microRNA200, and the upregulation of ZEB1 and BMP4 associated with epithelial to mesenchymal transition (EMT) program also render increased expression of PD-L1 on lung cancer cells in mice and humans^8^,^9^ Thus, PD-L1expression is regulated by both tumor intrinsic and tumor extrinsic pathways. More importantly, by using PD-L1 knockout mice and multiple PD-L1 knockdown or knockout tumor cell lines, we further showed that although PD-L1 was also highly expressed on tumor infiltrating myeloid cells and other stromal cells in the tumor microenvironment, it was the tumor cell-associated PD-L1 expression detected T cell exhaustion and immune suppression inside tumor tissues^9^. This result is consistent with the majority of data now published from clinical studies showing that the response rate and outcome of anti-PD-1/PD-L1 therapies correlate well with PD-L1 expression levels on tumor cells^1^,^2^,^4^. Taking consideration of all these findings and the fact that human PD-L1 can interact with mouse PD-1, we conceived an idea of constructing a simple human PD-L1 replacement mouse tumor model system for evaluating the functional consequence of blocking PD-L1 expressed on tumor cells without altering its presence on non-tumor cells.

Human peripheral lymphoid cells^10^,^11^,^12^, hematopoietic stem cells (HSC)^13^ or fetal liver cells^14^ were transferred into newborn or adult immuno-deficient mouse to construct humanized mouse model for pre-clinic screening of monoclonal antibodies which targeted to human immune checkpoint. These models have shown tremendous values in pre-clinic screening of antibodies. However, more and more researchers are reluctant to widely use these models for drugs screening by these limitations including high time- and economic-cost. Based on these considerations, we constructed a human PD-L1 replacement MC-38 tumor model for pre-clinic screening of immune checkpoint inhibitors targeted to human PD-L1. We first used CRISPR-Cas9 system to delete mPD-L1 and then expressed hPD-L1 in these mPD-L1 deletion cells^15^,^16^.

In this study, we constructed an hPD-L1 expressing MC-38 tumor animal model and observed an evident anti-tumor effect by treating with MPDL-3280A, the hPD-L1 monoclonal antibody. Flow cytometry analysis revealed antibody treatment increased the frequency and the cytotoxicity of infiltrated CD8^+^CTLs and repressed Treg cell enrichment, as well as facilitated the expansion of tumor infiltrating myeloid cells in tumor tissues. Taken together, we construct an hPD-L1 tumor animal model and we hope this model could contribute to quick pre-clinic antibody screening. Additionally, this model could also be used for pre-clinic screening of other immune checkpoint inhibitors.

## Results

### Replacement of mouse PD-L1 with human PD-L1 on MC-38 mouse colon cancer cells

**Figure 1:**
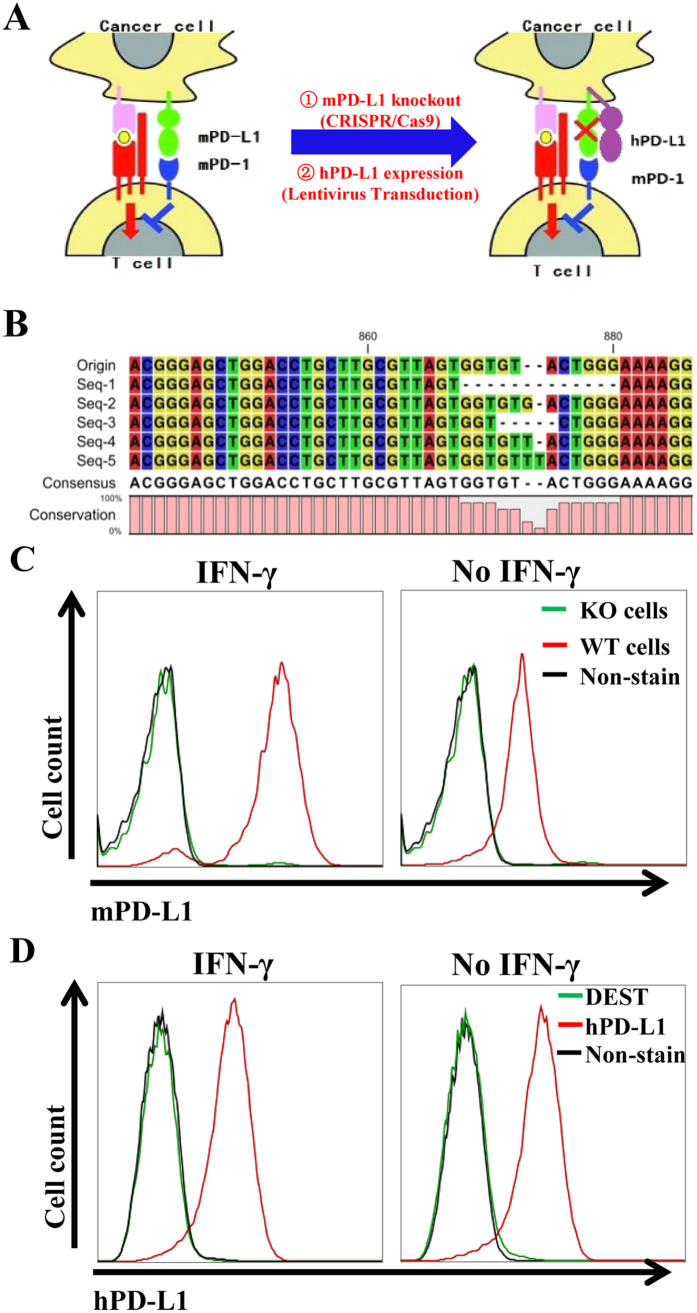
Humanized PD-L1 expression in MC-38 cell line. (**A**) Illustration of the principle and experimental design for mPD-L1 knockout (by CRISPR/Cas9 system) and replacement with hPD-L1 expression (by lentivirus transduction). (**B**) Confirmation of mPD-L1 knockout in MC38 cells (KO) by genomic DNA sequencing and frameshift Indel analsyis. (**C**) Flow cytometry analysis of mPD-L1 expression on MC-38 and MC-38 KO cells with or without IFN-γ stimulation. (**D**) Flow cytometry analysis of hPD-L1 expression on MC-38 KO DEST (vector control) and MC-38-hPD-L1 cells with or without IFN-γ stimulation. Non-staining cells act as control.

Multiple recent studies showed that PD-L1 which was highly expressed on almost all types of cancer, but not tumor stromal or other host cells plays essential role in suppressing antitumor immunity and driving tumor growth in a number of different tumor settings^9^. There are few effective and inexpensive pre-clinic animal tumor models to screen antibodies which target to hPD-L1. Therefore, to construct a simple and straightforward strategy to screen the therapeutic efficacy of anti-human PD-L1 antibodies *in vivo* is in favor with the general public. This study reported a human PD-L1 tumor model which was constructed by replacing mouse PD-L1 with human PD-L1 in a common transplantable mouse tumor cells (MC-38 cells, Fig. 1A). Firstly, we completely deleted mPD-L1 on MC-38 cells by CRISPR-Cas9 system, and the deletion efficiency was examined by sequencing (Fig. 1B) and flow cytometry (Fig. 1C). We next infected these mPD-L1 deleted MC-38 cells (MC-38 KO) with hPD-L1 expressed lenti-virus. Flow cytometry revealed hPD-L1 was highly expressed in MC-38 KO cells with or without IFN-γ stimulation (Fig. 1D). Taken together, we completely deleted mPD-L1 on MC-38 cells and subsequently constructed an hPD-L1 replaced MC-38 cell line.

### hPD-L1 expressing MC-38 tumor model was generated

**Figure 2:**
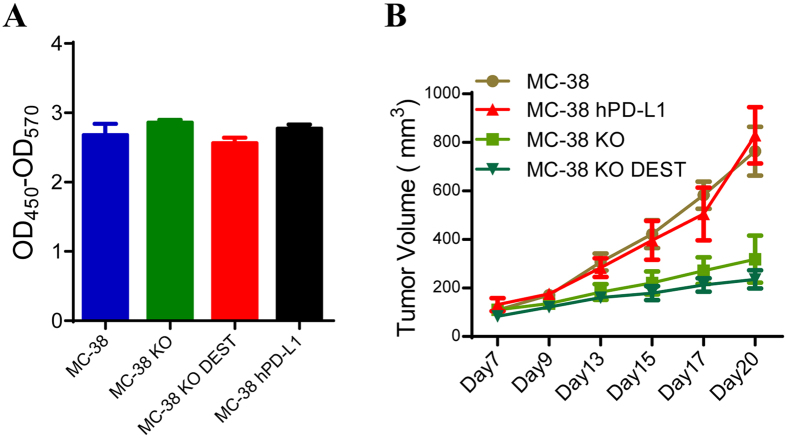
Generation of hPD-L1 replacement MC-38 tumor animal model. (**A**) The proliferation activity of hPD-L1 replacement MC-38 cells (MC-38 hPD-L1) *in vitro* (n = 4). (**B**) Tumor growth of MC-38 hPD-L1 tumors in a 2.0*10^6 inoculation (n = 8). MC-38 KO means mouse PD-L1 KO, MC-38 KO DEST means empty vector for human PD-L1 over-expressing. All quantitative data are represented as means ± SEM.

It is unclear whether hPD-L1 replacement affected tumor cell growth *in vitro* and vivo. To address these questions, we examined the growth of MC-38, MC-38 KO, MC-38-DEST (vector control), MC-38-hPD-L1 cells *in vitro* (Fig. 2A). CCK8 results indicated mPD-L1 deletion or hPD-L1 replacement did not intrinsically suppress MC-38 cell growth *in vitro*. These cells was subcutaneously inoculated into C57BL/6 mouse for tumor model construction. Tumor volume was detected every two days when the tumor volume reached 100 mm^3^ (Fig. 2B). As expected, tumor growth in mouse inoculated with mPD-L1 KO cells was significantly repressed. More importantly, the growth of hPD-L1 replacement tumors was almost similar to MC-38 tumors. Therefore, we conclude hPD-L1 MC-38 tumor model was successfully constructed.

### Anti-hPD-L1 antibody treatment reduced human PD-L1 tumor growth

**Figure 3:**
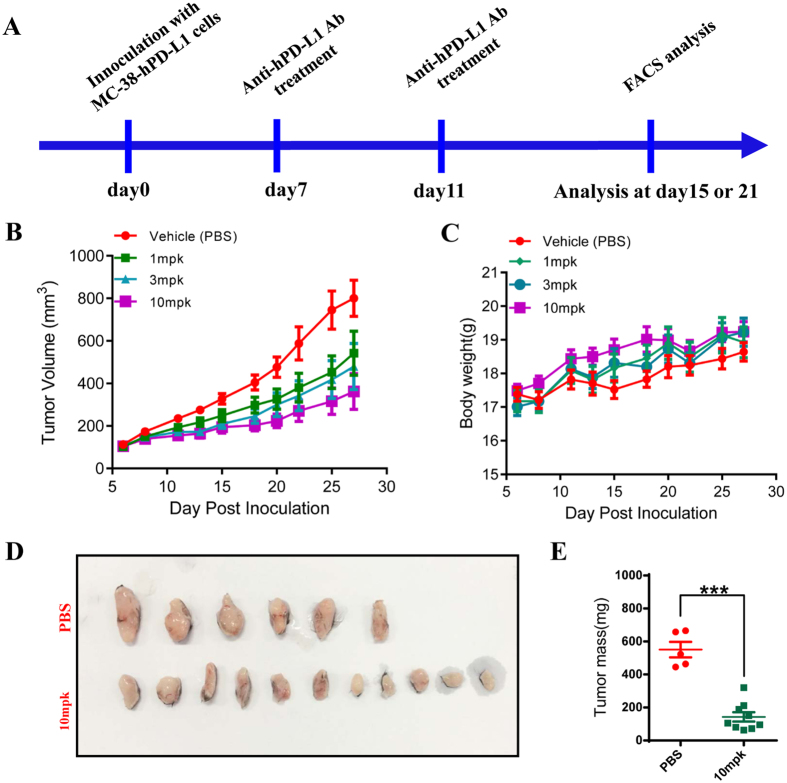
Anti-human PD-L1 antibody treatment inhibits MC-38 hPD-L1 tumor growth. (**A**) Experimental scheme of MC-38 hPD-L1 tumor inoculation and anti-human PD-L1 antibody treatment. Tumor growth (**B**) and body weight (**C**) of the tumor bearing mice treated with different dose of anti-human PD-L1 antibody (MPDL-3280A) (n = 8). Image of subcutaneous tumor mass (**D**) and tumor weight (**E**) of MC-38 hPD-L1 tumors treated with 10mpk of anti-hPD-L1 antibody. All quantitative data are represented as means ± SEM, ***denotes p < 0.001.

To determine whether this model could be used for screening antibodies which targeted to hPD-L1, we subcutaneous inoculated MC-38-hPD-L1 cells into C57BL/6 mouse and treated these tumor bearing mice with anti-hPD-L1 antibody by intra-peritoneal manner. Additionally, we first determined the binding of human PD-L1 (hPD-L1) to mouse PD-1 (mPD-1) by ELISA. The OD value indicated that the hPD-L1 bound to mPD-1 very well (Supplementary Fig. 1). Furthermore, the binding of human PD-L1 to mPD-1 was also confirmed in MC-38-hPD-L1 cells by using flow cytometry analysis (Supplementary Fig. 2). The phenotype of tumor infiltrated lymphocytes was measured by flow cytometry analysis at day 15 and day 21 after inoculation (Fig. 3A). As expected, anti-hPD-L1 antibody treatment remarkably reduced tumor growth at just 1.0 mpk dose (Fig. 3B,C). The tumor weight of anti-hPD-L1 antibody treated group was remarkably reduced (Fig. 3D,E). We sacrificed all mice 21 days after tumor implantation for subsequently flow cytometry analysis, and there was no death of the treated animals. Taken together, our results indicated treatment with anti-hPD-L1 antibody in this model was effective and safety.

### Anti-hPD-L1 antibody treatments promte T cell infiltration but reduce Tregs enrichment

**Figure 4:**
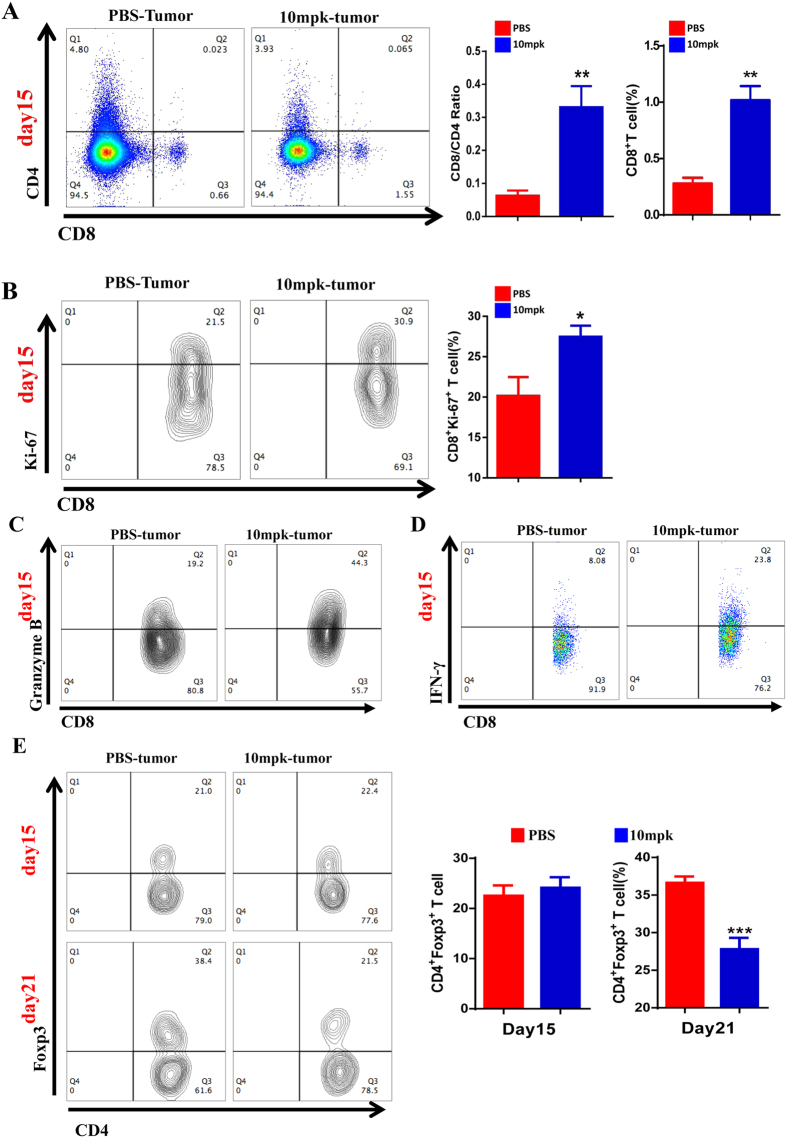
Phenotype of tumor infiltrating T cells in MC-38 hPD-L1 tumor bearing mice was reversed by anti-hPD-L1 antibody therapy. (**A**) Flow cytometry analysis of infiltrating CD4^+^/CD8^+^T cell (n = 5) frequency with or without anti-hPD-L1 antibody treatments. (**B**) Flow cytometry analysis of tumor infiltrating CD8^+^Ki-67^+^T cell (n = 3) frequency after anti-hPD-L1 antibody therapy. Flow cytometry analysis of Granzyme B (**C**) and IFN-γ (**D**). (**E**) Determination of tumor infiltrating CD4^+^Foxp3^+^Treg cells phenotype at day15 (n = 3) and day21 (n = 9) by FACS with or without anti-hPD-L1 antibody treatments. All quantitative data are represented as means ± SEM, *denotes p < 0.05, **denotes p < 0.01, ***denotes p < 0.001.

Many clinic data have reported that PD-L1 blocking promotes CD8^+^T cell infiltration. To address whether this model also hit this point, the phenotype of immune cells in tumor microenvironment were analyzed by flow cytometry. The frequency of CD8^+^T cells and the ratio of CD8 to CD4 T cells were both remarkably up-regulated (Fig. 4A). The phenotype of T cells in mPD-L1 KO tumors was also examined. Consistently, mPD-L1 KO significantly promoted CD8^+^T cell infiltration (Supplementary Fig. 3). In addition, we observed that anti-hPD-L1 antibody treatment also impaired the balance of CD4 to CD8 T cells in splenocytes (Supplementary Fig. 4). The frequency of tumor infiltrated or peripheral blood T cells was also examined by flow cytometry with two treatment manners: multiple course of antibody treatments or single antibody treatment (Supplementary Fig. 5). Furthermore, the level of PD-1 in infiltrated T cells was also examined (Supplementary Fig. 6). As we expected, flow cytometry analysis indicated that the frequency of CD8^+^T cells arised in single course anti-hPD-L1 antibody treatment (Supplementary Fig. 7). However, the frequency of CD8^+^T cells did not shown any difference in peripheral blood (Supplementary Fig. 8). Consistently, CD8^+^T cell was significantly increased in tumors but not peripheral blood in multiple course antibody treatment groups (Supplementary Fig. 9).

To examine the cytotoxicity of tumor infiltrated CD8^+^T cells, these T cells were subjected to flow cytometry analysis for examining the level of ki-67, Granzyme B and IFN-γ. As expected, the level of ki-67 (Fig. 4B), Granzyme B (Fig. 4C) and IFN-γ (Fig. 4D) in CD8^+^CTLs were all increased in anti-hPD-L1 antibody treated mice. Extensively, the IFN-γ level were also tested by Enzyme-linked Immunospot (ELISPOT) assay (Supplementary Fig. 10). Consistently, IFN-γ expression was significantly increased in anti-hPD-L1 antibody treated tumors. Moreover, the Tregs frequency in tumors was not altered by anti-hPD-L1 antibody treatment at day15 but significantly reduced at day21 (Fig. 4E). To summarize these data, we conclude that antibody treatment significantly increases CD8^+^CTLs infiltration but suppresses Tregs enrichment in tumor microenvironment.

### Anti-hPD-L1 antibody treatments prompt myeloid cell recruitment to tumor microenvironment

**Figure 5:**
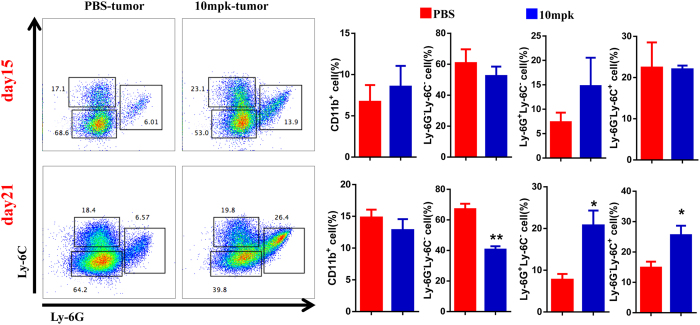
Antibody therapy altered the phenotype of tumor infiltrating myeloid cells. Flow cytometry analysis of tumor infiltrating CD11b^+^Ly-6C/Ly-6G^+^populations of myeloid cells with or without anti-hPD-L1 antibody treatment (10 mg/kg) at day15 (n = 3) and day21 (n = 9). All quantitative data are represented as means ± SEM, *denotes p < 0.05, **denotes p < 0.01.

We also asked whether anti-hPD-L1 antibody affected recruitment of tumor infiltrated myeloid cells. To address this question, we analyzed CD11b^+^, Ly6G^+^or Ly6C^+^myeloid cells in tumor tissues by flow cytometry. FACS data revealed CD11b^+^myeloid cells were not affected by anti-hPD-L1 antibody treatment. Amazingly, the frequency of CD11b^+^Ly6G^+^cells was slightly increased at day15 but significantly increased at day21. Similarly, though the frequency of CD11b^+^Ly6C^+^was not affected at day15, it was significantly increased at day21 (Fig. 5). To confirm these results, we also analyzed the frequency of these cell types in mPD-L1 KO tumors. Consistently, the frequency of these two myeloid subsets was all increased in mPD-L1 KO tumors (Supplementary Fig. 11). Furthermore, the level of PD-1 in myeloid cells was also measured by flow cytometry (Supplementary Fig. 6). Taken together, these results suggested anti-hPD-L1 antibody treatment promotes myeloid cells recruitment.

## Discussion

In this study, an hPD-L1 tumor animal model was established. We expected this model could make few contributions for anti-hPD-L1 monoclonal antibody pre-clinic screening. As the results showed, we used genetic engineering methods to replace mouse PD-L1 by human PD-L1 in MC-38 cells. To explore whether this model was available to antibody screening, we treated human PD-L1 replacement MC-38 cells with anti-hPD-L1 antibody, a verified human PD-L1 antibody. In this model, anti-hPD-L1 antibody remarkably suppressed the growth of tumors and also increased the frequency and the cytotoxicity of CD8^+^CTLs and CD11b^+^Ly-6G^+^/6 C^+^monocytes in tumors. Besides of these findings, treatments with anti-hPD-L1 antibody significantly reduced Tregs infiltration. Rapid development of CRISPR-Cas9 technology promotes us to quickly construct humanized tumor cells. Though we selected PD-L1 as the candidate here, this model could be used for screening antibodies for other immune checkpoints before clinical trials. Furthermore, the success of this workable model facilitates us to generation new humanized models for other antibodies screening such as OX40 L, 4-1BBL or CD40.

In this study, we notified that anti-hPD-L1 antibody treatment enhanced the frequency and cytotoxicity of CD8^+^infiltrated T cells. It is not very clear how anti-PD-L1 antibody increases the frequency of infiltrating CD8^+^CTLs. Antibody treatment either promotes CD8^+^T cells recruitment into tumor^17^,^18^ or prolong the survival of CTLs^19^. Though PD-L1 blocking increased CD8^+^T cell frequency and CD8^+^T to CD4^+^T cells ratio in this human PD-L1 tumor model, we need take tumor volume in consideration. The respected cell populations per mm2 of tumor size would also be a more accurate way of measuring the actual increase of immune cells. However, it is a general problem to consistently and faithfully count total number of live cells extracted out from tumor tissues, therefore measurement of absolute cell numbers is not reliable, and on the other hand, relative percentage of each population of cells can be readily obtained and is commonly used by many research groups showing good correlation with anti-tumor activity inside tumor tissues. For the further investigation, we would use immunohistochemistry analysis for detecting the actual increase of these immune cells. Additionally, depletion of CD8^+^T cells by anti-CD8 antibody treatment would be a sensible experiment to directly address the requirement of CD8^+^CTLs for the antitumor function of anti-human PD-L1 therapy.

Regulatory T cells refer to diverse populations of lymphocytes that display inhibitory and regulatory function by regulating the activity of immune cells in tumor microenvironment. More and more results indicate immune checkpoint blocking may inhibit Tregs infiltration. CTLA-4 blocking engage on CD16 expressing, non-classical monocytes resulting in ADCC-mediated lysis of regulatory T cells^20^. Consistent with that, PD-L1 blockade suppressed Tregs frequency in our human PD-L1 tumor model. On the other hand, reduced Tregs liberated CD8 CTLs from cytotoxic suppression. This must be the positive factors for tumor clearance in this human tumor model.

Recently, multiple studies have reported that myeloid derived cells contributed to tumorigenesis. In this study, we also explored the effects of PD-L1 antibody treatment on myeloid cell recruitment. We have observed a consistent increase of CD11b^+^Gr1^+^myeloid cell populations inside tumor tissues after anti-PD-L1 treatment in our model, and similar results were also found in our previous studies^9^. The CD11b^+^myeloid cells are composed both of Ly6C^+^Ly6G^−^ and Ly6C^−^Ly6G^+^, as well a minor Ly6C^+^Ly6G^+^subpopulation. It remains unclear whether some of these cells are MDSCs of suppressive function, while others present inflammatory monocytes, M1 macrophages and neutrophils which can facilitate anti-tumor response of CD8^+^CTLs, and the eventual tumor clearance. *In vivo* depletion of some of the subpopulations by anti-Gr1 and/or anti-Ly-6G antibody treatment would help to delineate their causative relationship with the anti-tumor efficacy mediated by anti-PD-L1 therapy. Moreover, disintegrating tumor cells released stress factors could recruit CD11b^+^Ly-6G^+^/Ly-6C^+^myeloid cells into tumors in an ATP^21^ or CCL2^22^ dependent manner and act as a antigen present cells (APCs). Recently, the ability of myeloid cells to promote Tregs differentiation has been described^23^,^24^. These paradox should been extensively explored in our future research. Combination to our data, we suspect that the expansion of CD11b^+^Ly-6G^+^/Ly-6C^+^myeloid cells is the response of tumor cells disintegration. Though CD11b^+^Ly-6G^+^/Ly-6C^+^myeloid cells contributes to Tregs differentiation, PD-L1 blocking plays vital role in suppressing the recruitment of Tregs in this tumor model. This complicated hazes need to be confirmed by more experimental results, but it does not disturb us to make an effective model to pre-clinic screening of anti-hPD-L1 antibodies.

## Materials and Methods

### Ethical considerations

All experiments were approved by the institutional ethics board (Chinese Academy of Medical Sciences). All experiments were carried out in accordance with the approved guidelines.

### Generation of PD-L1 CRISPR/Cas9 knockout mouse tumor cell line

C57BL/6 derived mouse colon tumor cell line MC-38 was originally obtained from ATCC (ATCC#CRL-2638). Short-guide RNAs (sgRNA) specific to mouse PD-L1 (CD274) was designed according to online design tool (http://crispr.mit.edu), cloned into sgRNA expression vector (FG-BB-U6-sgRNA) at BbsI restriction site, and confirmed by sequencing. CRISPR/Cas9 knockout was performed by co-transfection of the anti-mPD-L1 sgRNA construct with Cas9 expression plasmid (FG-hEF/HTLV-Cas9-PGK-Puro-WPRE) carrying a puromycin selection marker and into the parental MC-38 cells using Lipofectamine 3000 (Invitrogen), and the sgRNA sequence is 5′-GCTTGCGTTAGTGGTGTACT-3′. The transfected cells were transient selected with puromycin for 2 days, then expanded and cloned to screen for target gene knockout by flow cytometry and TA cloning-sequencing. For TA cloning-sequencing analysis, genomic DNA was extracted from the pooled cells by DNeasy Blood&Tissue kit (Qiagen), forward primer is 5′-TGGTTCCTTTTAAACAAGACTGGG-3′, and reverse primer is 5′-CGCACCACCGTAGCTGATTA-3′. The sgRNA targeted DNA fragment was amplified by Taq DNA polymerase (NEB) and cloned into pTZ57R vector using Ins TAclone PCR Cloning Kit (Fermentas Thermo). Numbers (5 clones) of individual resulting TA plasmid clones were sequenced, and the sequencing results were analyzed by ClusterW software. Frameshift Indels in mouse PD-L1 gene were identified by Blast (NCBI), and functional knockout was confirmed by flow cytometry for the absence of PD-L1.

### Construction of hPD-L1 replacement mouse tumor cell line

Lentiviral expression vector (FG-hEF/HTLV-human CD274-PGK-Puro-WPRE) carrying human PD-L1 ORF was constructed and verified by sequencing. High titer recombinant lentivirus particles were produced from HEK293T cells by calcium phosphate transfection with the third generation of lentiviral packaging plasmids according to Qin *et al*.^25^. The mPD-L1 knockout MC-38 cells were then transduced with the hPD-L1 expressing lentivirus and selected by Puromycin to establish the stable hPD-L1 replacement MC-38 cell line (MC-38 hPD-L1). Successful replacing mPD-L1 by hPD-L1 was confirmed by flow cytometry analysis.

### Human PD-L1 to mouse PD-1 binding assay

The plate was coated with mPD-L1-Fc or hPD-L1-Fc protein in high pH coating buffer overnight at 4 °C. Added diluted biotin labeled mPD-1 protein into each well with dilution buffer for 1 h at room temperature after incubated by blocking buffer. Added diluted Neutavidin-HRP (1:5000 dilution) to wells and incubated for 1 hr in room temperature. Read OD value of the plate at 450 nm by automatics plate reader (Molecular Devices, USA).

We performed flow cytomtry analysis to determine whether mouse PD-L1 could bind to human PD-L1. Briefly, 1.0*10^6 MC-38-hPD-L1 cells were incubated with 5.0 μg/ml or 2.5 μg/ml mouse PD-1 protein for 25 min after the cells were blocked by FcR antibody. Next, these cells were washed by staining buffer for 3 times and then stained by anti-Fc-APC for 20 min. These cells were subjected to flow cytometry analysis. All of these proteins and antibodies are obtained from Mabspace Biosciences (Suzhou) Co., Ltd.

### In vitro cell proliferation and viability analysis

Cell proliferation activity was determined by Cell Counting Kit 8 (CCK8) purchased from Beyotime (Shanghai, China) according to the manufacturer’s instruction. Briefly, the 96 well plate was seeded with 20,000 cells per well and cultured in 100 μl 10% FBS RPMI-1640 medium for 24 h. We next added 10 μl CCK8 reagent and incubated for 4 h. The plate was measured by automatics plate reader (Molecular Devices, USA).

### Animal model and tumor treatment

Female C57BL/6 mice were purchased from Vital River Laboratories (Beijing, China). All mice were maintained in a barrier facility at Soochow University. To establish subcutaneous tumor, C57BL/6 mice were inoculated with various tumor cell lines in the right side axillae with 0.2 mL/mouse of the cells adjusted to 1.0 × 10^7^ cells/mL (2 × 10^6^ cells/mouse). Tumor growth was monitored and measured every two days using digital caliper (INSIZE, Austria), and the tumor volume was calculated according to the following formula: M1^2^*M2 /2 (M1: short diameter; M2: long diameter). When the tumor volume reached 100 mm^3^ (day 7), mice were treated with 1.0 mpk, 3.0 mpk or 10.0 mpk of anti-hPD-L1 antibody (the antibody was synthesized by MabSpace Biosciences (YW243.55.S70 or MPDL3280A as disclosed in the US Patent No. 8217149) or PBS vehicle control by intraperitoneal (i.p.) injection, and the second dose of treatment was given 4 days later. The mice were sacrificed for immune phenotype and function analyses either 4 or 10 days after the second dose of treatment.

### Flow cytometry

Flow cytometry was performed with Life Attune Nxt FACS machine (Life Technology/Thermo Fisher Scientific, Waltham, MA). Fluoro-chrome conjugated anti-mouse CD45 (30-F11, Biolegend), CD3 (145–2C11, eBioscience), CD4 (RM4-5, eBioscience), CD8 (eBioH35-17.2, eBioscience), Foxp3 (FJK-16s, eBioscience), Ki-67 (SolA15, eBioscience), Granzyme B (NGZB, eBioscience), Ly-6C (AL-21, Biolegend), Ly-6G (1A8, Biolegend), mPD-L1 (10 F.9G2, Biolegend), hPD-L1 (29E.29A3, Biolegend) and IFN-gamma (XMG1.2). Granzyme B and IFN-gamma staining was performed with intracellular staining kit from BD Bioscience. While Ki-67 and Foxp3 staining was done with nuclear protein staining kit from eBioscience (San Diego, CA). FACS data were analyzed using FlowJo software (Tree Star, Ashland, OR).

### Enzyme-linked immunospot assay (ELISPOT)

The IFN-γ ELISPOT assay was performed according to manufacturer’s protocol from BD life science. Briefly, the ELISPOT plate was coated with anti-mouse IFN-γ capture antibody diluted in PBS at 5.0 μg/ml overnight at 4 °C. 2.0*10^5^ tumor tissue cells were added to the plate per well in 100 μl and then incubated the plate at 37 °C for 12 h in a 5% CO_2_ incubator. Discard the cell suspension and wash every micro-well by Elispot wash buffer. Added the diluted detection antibody at 2.0 μg/ml in 100 μl and incubated the plate at room temperature for 2 h. After washing with PBS, the plate was incubated with 100 μl streptavidin-HRP conjugate for 1 h at room temperature. Then washed the plate for 3 times by Elispot by wash buffer and added 100 μl final substrate solution for antibody detection. Stop the substrate reaction by washing wells with DI water. Enumerate spots of every micro-well by automatical ELISPOT plate reader (Celluar Technology Ltd. USA).

### Statistical analysis

Statistical analysis was performed for all experiments using Student’s t-test. Values of p inferior to 0.05 were considered significant. All tests were done using Prism 6 software (Graph Pad Prism6).

## Additional Information

**How to cite this article**: Huang, A. *et al*. A human programmed death-ligand 1-expressing mouse tumor model for evaluating the therapeutic efficacy of anti-human PD-L1 antibodies. *Sci. Rep.* **7**, 42687; doi: 10.1038/srep42687 (2017).

**Publisher's note:** Springer Nature remains neutral with regard to jurisdictional claims in published maps and institutional affiliations.

## Supplementary Material

Supplementary Information
