# Supplementary material for: A human programmed death-ligand 1-expressing mouse tumor model for evaluating the therapeutic efficacy of anti-human PD-L1 antibodies
Source: Sci Rep. 2017 Feb 16;7:42687. doi: 10.1038/srep42687 (PMC5311961; doi:10.1038/srep42687)
Supplement: Supplementary Information [file srep42687-s1.pdf]

A human programmed death-ligand 1-expressing mouse tumor model for evaluating the therapeutic efficacy of anti-human PD-L1 antibodies

Anfei Huang[1,#], Di Peng[1,#], Huanhuan Guo[2], Yinyin Ben[1], Xiangyang Zuo[1], Fei Wu[1], Xiaoli Yang[3], Fei Teng[2], Zhen Li[2], Xueming Qian[3], F. Xiao-Feng Qin[1,*]

[1] Center of Systems Medicine, Institute of Basic Medical Sciences, Chinese Academy of Medical Sciences & Peking Union Medical College, Beijing, 200005, China, Suzhou Institute of Systems Medicine, Suzhou, 215123, China.

[2] Mabspace Biosciences (Suzhou) Co., Ltd, Suzhou, 215123, China.

[3] Institutes of Biology and Medical Sciences, Soochow University, Suzhou, 215123, China.

[#] Anfei Huang and Di Peng contributed equally to this work.

[*]Correspondence should be addressed to F. Xiao-Feng Qin (fqin1@foxmail.com).

Supplementary Fig.1. The binding activity of hPD-L1/mPD-L1 to mPD-1. The plate was coated with mPD-L1-Fc(Green) or hPD-L1-Fc(Red) protein, and the binding activity to mPD-1 was detected at 450nm after incubated by Neutavidin labeled HRP. Non-coat plates was considered as the control (Black).

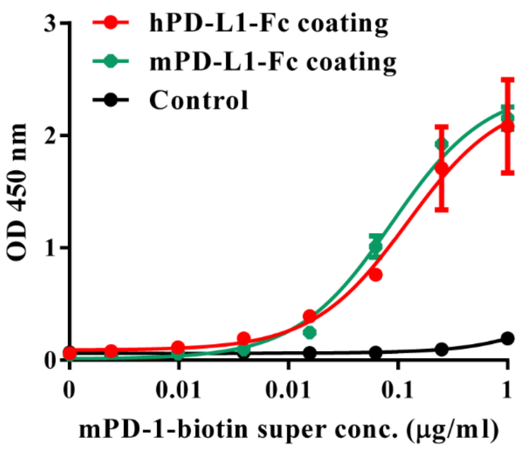

Supplementary Fig.2. The binding activity of hPD-L1 to mPD-1 was detected by flow cytometry. 1.0*10^6 MC-38-hPD-L1 cells were incubated with 5.0 μg/ml or 2.5 μg/ml mouse PD-1 (mPD-1) protein for 25 min after blocked with FcR antibody. The fluorescence intensity was analyzed by flow cytometry.

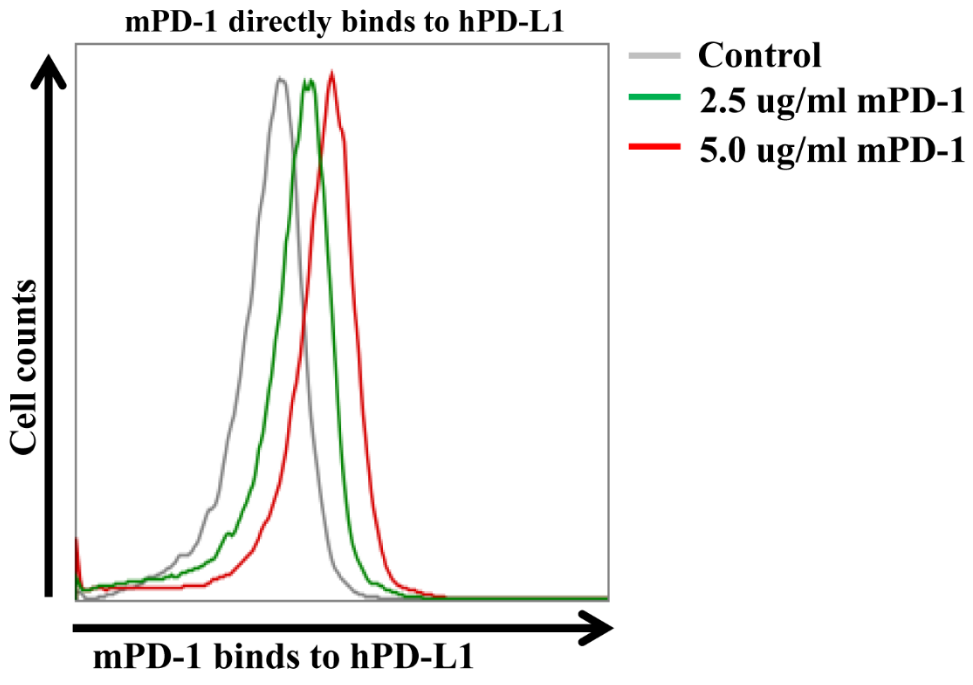

Supplementary Fig.3. T cell phenotype in mPD-L1 KO tumors. 2.0*10^6 MC-38-DEST (mPD-L1 KO) cells were inoculated and the tumor tissues were analyzed by flow cytometry at day 15.

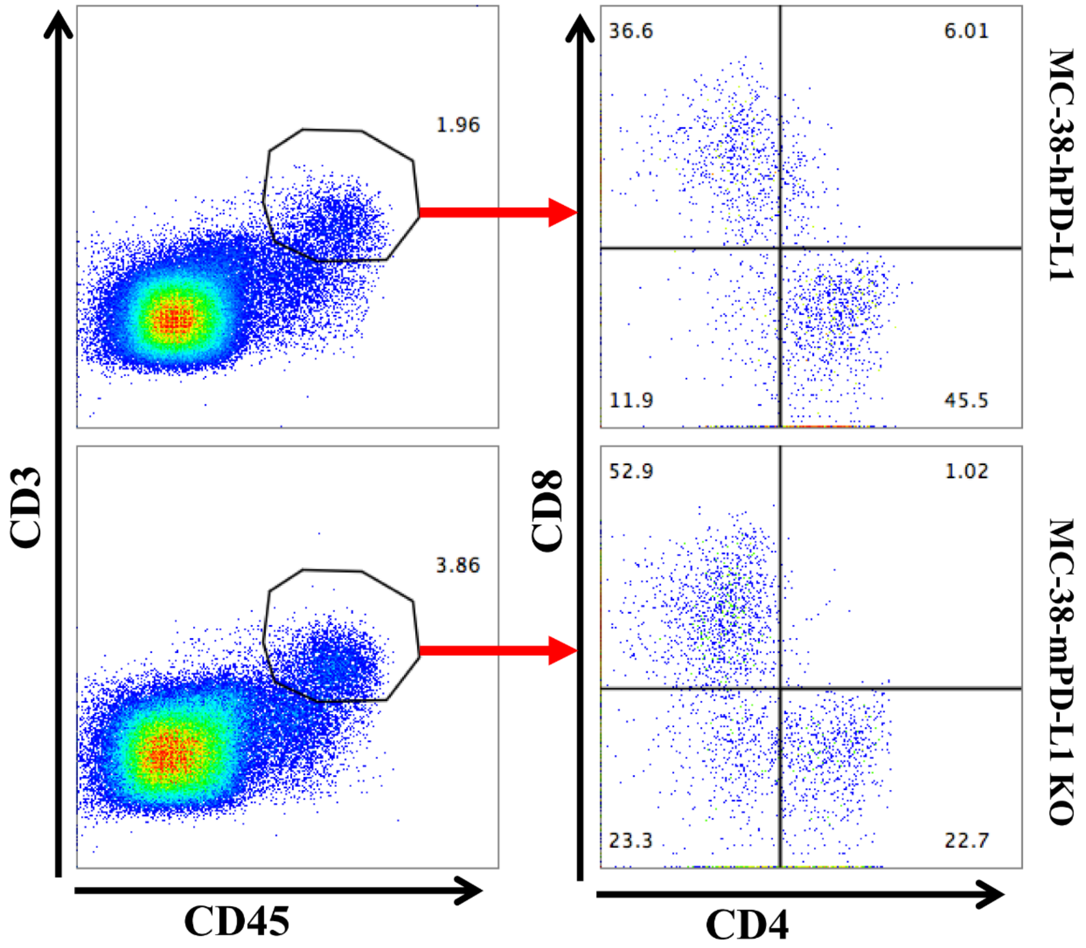

Supplementary Fig.4. T cell phenotype in splenocytes after PD-L1 antibody treatments. Flow cytometry analysis of T cell in splenocytes with the anti-hPD-L1 antibody treatment.

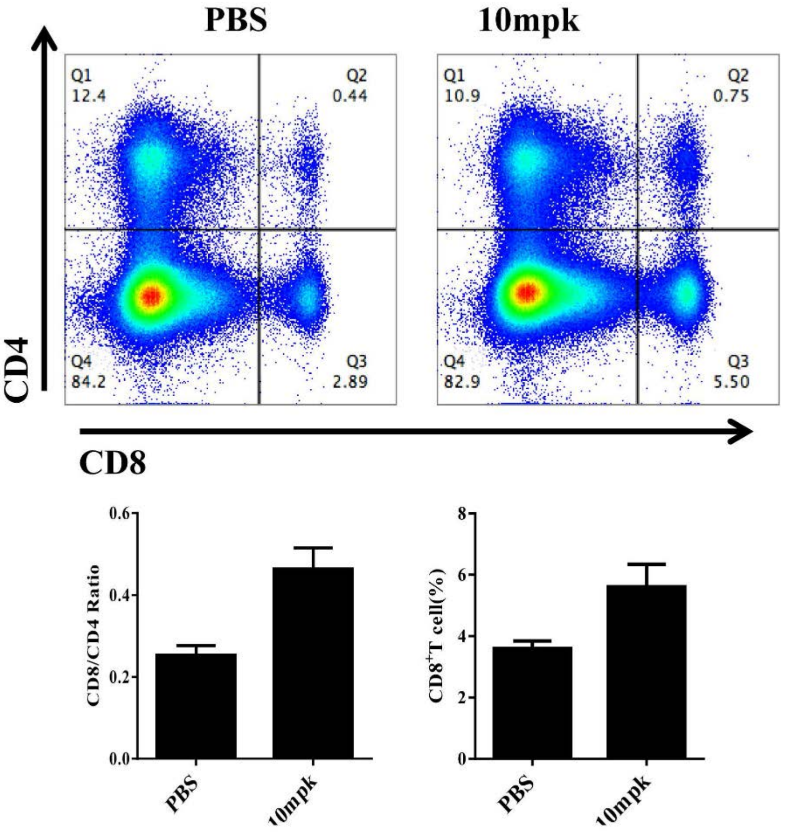

Supplementary Fig.5. The schedule of multiple or single course of antibody treatments.

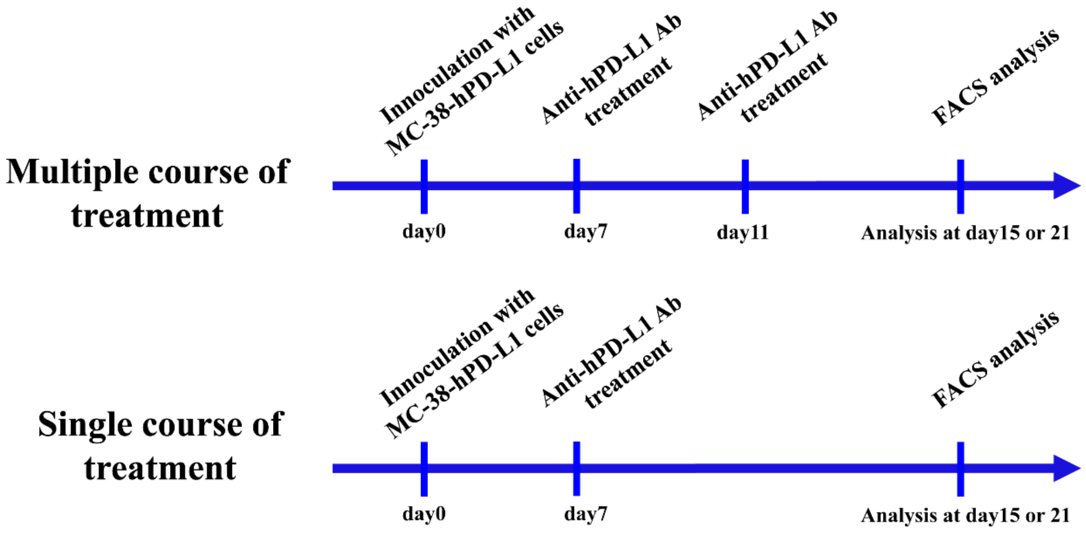

Supplementary Fig.6. The PD-1 expression in infiltrated T cells and myeloid cells. 2.0*10^6 MC-38-hPD-L1 cells were inoculated and the tumor tissues were analyzed by flow cytometry at day 15. CD4 or CD8 T cells gated on $CD45^+CD3^+$ cells. 10 mpk represents antibody treated group.

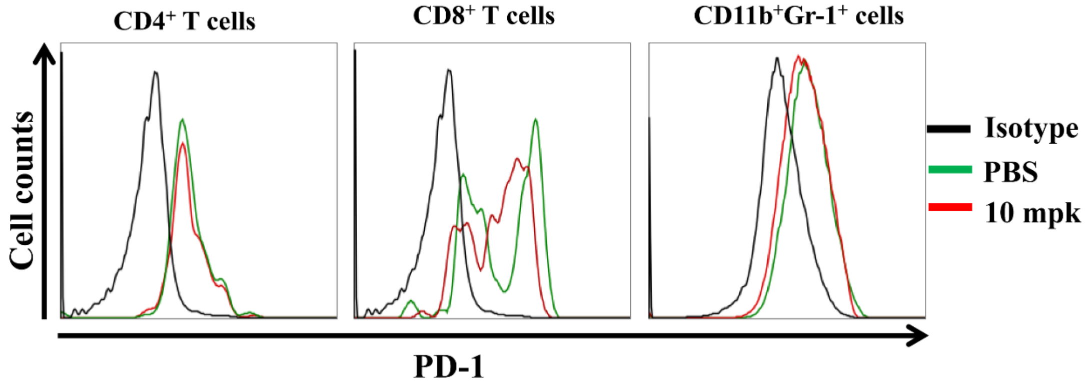

Supplementary Fig.7. The frequency of CD8$^+$ T cells in tumor tissues with anti-hPD-L1 treatments. Flow cytometry analysis of CD8$^+$T cell phenotype in tumor tissues at day3, 7, 10 after antibody treatment.

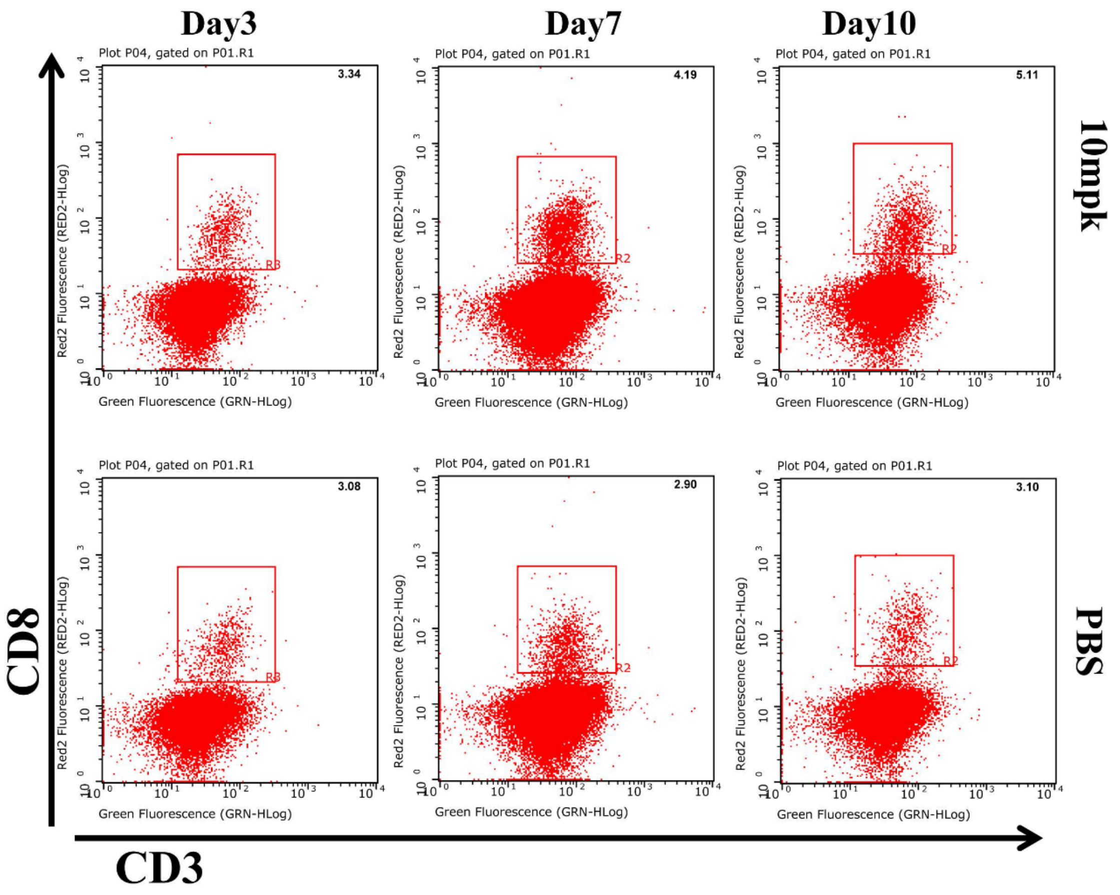

Supplementary Fig.8. The frequency of CD8$^+$ T cells in peripheral blood with anti-

hPD-L1 treatments. Flow cytometry analysis of CD8$^+$ T cell phenotype in peripheral blood at day 7, 10 after antibody treatment.

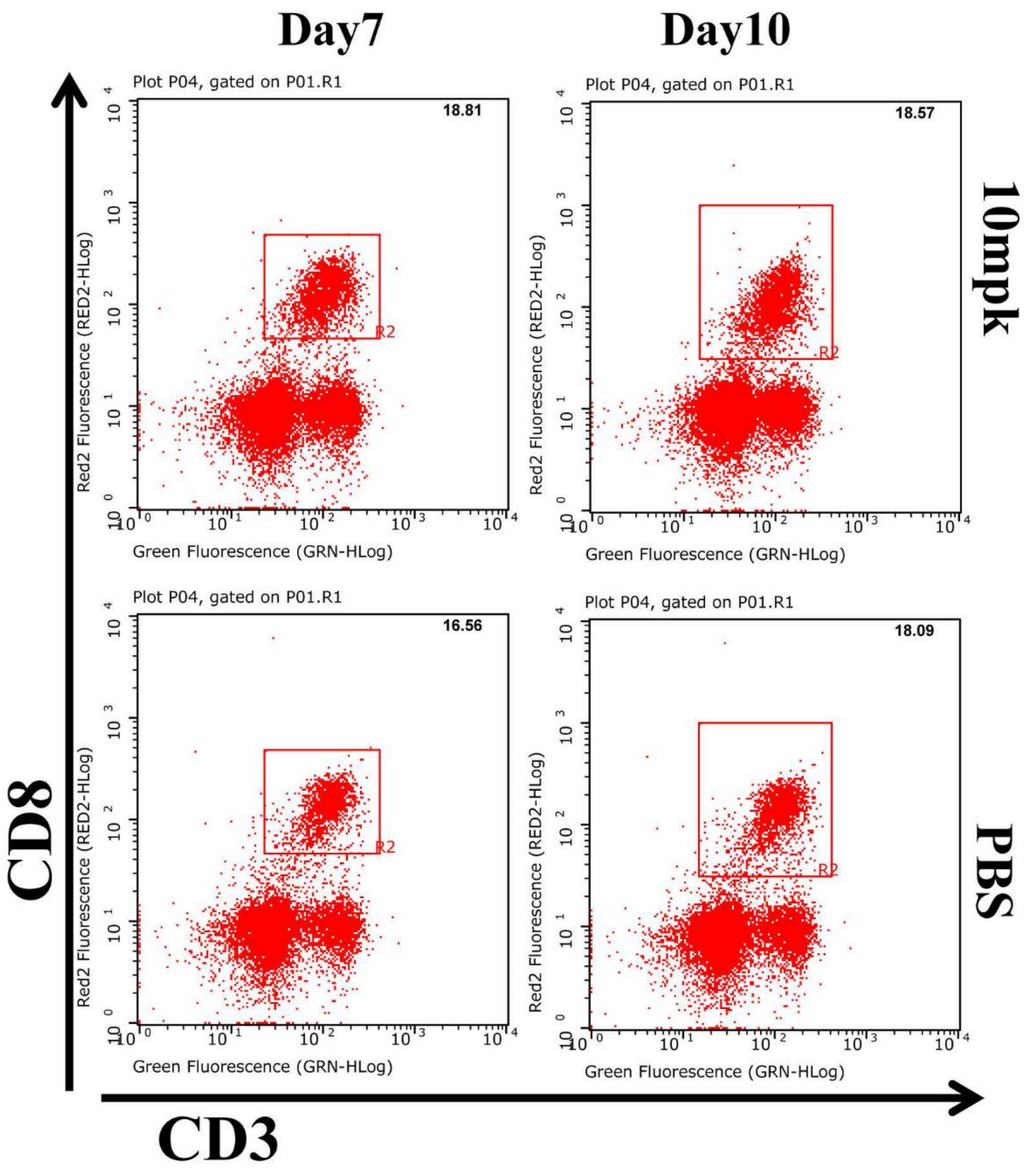

Supplementary Fig.9. The frequency of CD8$^+$ T cells in tumor tissues or peripheral in multiple course antibody treatment model. Flow cytometry analysis of CD8$^+$ T cell phenotype in tumor tissues(left) or PBMC from tumor bearing mouse(right) at day 10 after antibody treatment.

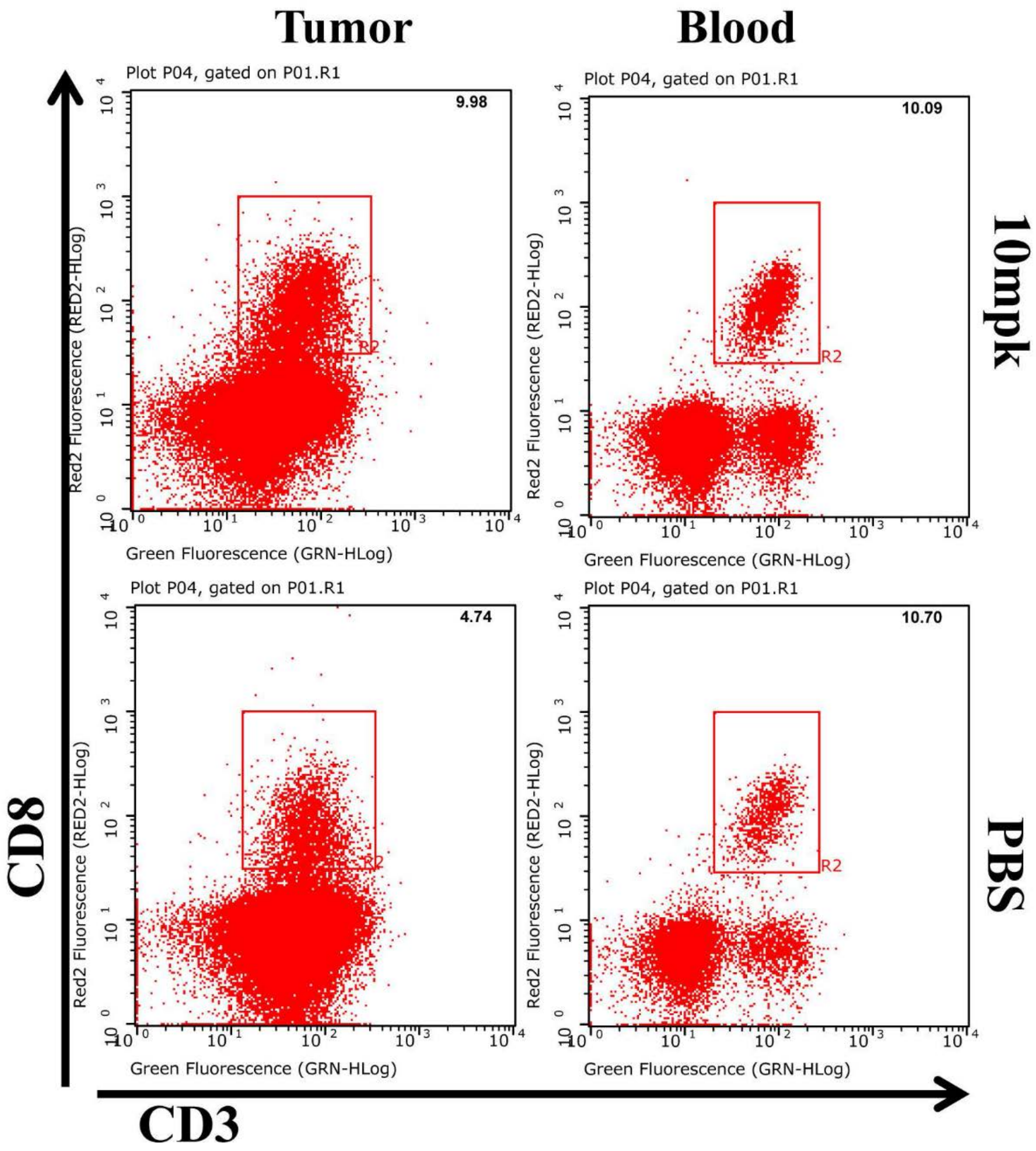

Supplementary Fig.10. Anti-PD-L1 antibody treatment promotes the expression of IFN-γ. The phenotype of $CD8^+$ T cells was analyzed by flow cytometry (left). The expression of IFN-γ in tumor tissue cells were detected by ELISPOT(right).

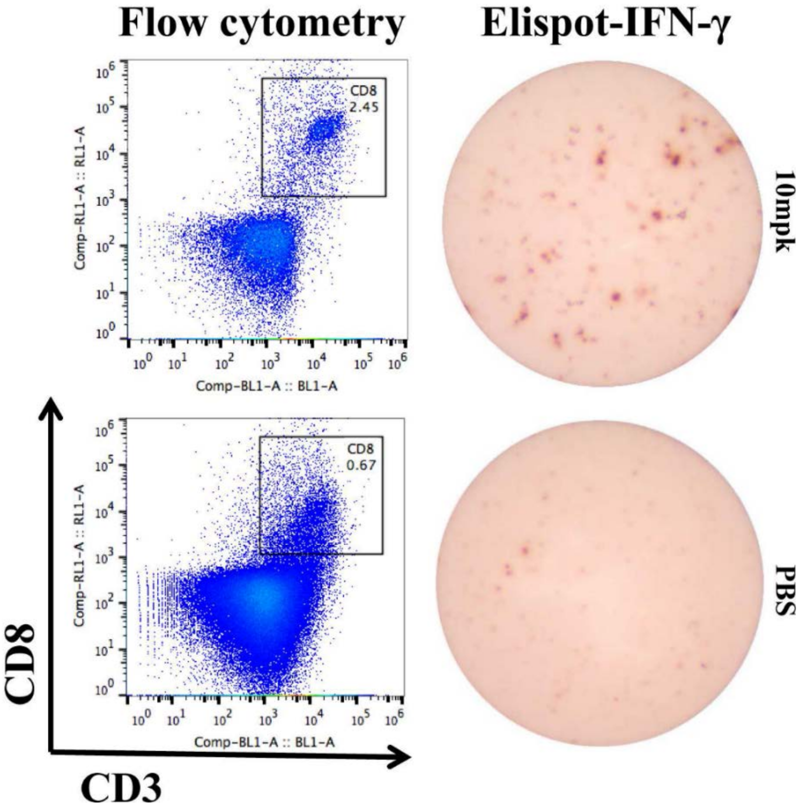

Supplementary Fig.11. The phenotype of infiltrated myeloid cells in mPD-L1 KO tumors. 2.0*10^6 MC-38-DEST (mPD-L1 KO) cells were inoculated and the tumor tissues were analyzed by flow cytometry at day 15. Cells were gate on CD11b$^+$ myeloid cells.

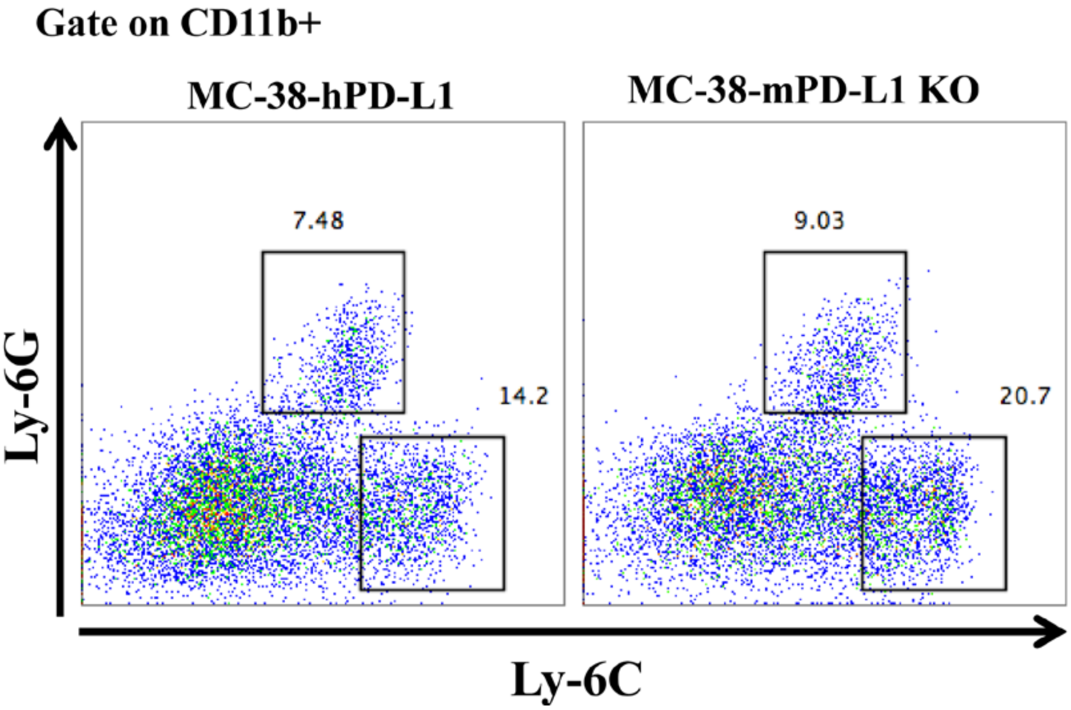
Gate on CD11b+
MC-38-hPD-L1
MC-38-mPD-L1 KO
7.48
14.2
9.03
20.7
Ly-6G
Ly-6C
